# Nutrition for Children with Down Syndrome—Current Knowledge, Challenges, and Clinical Recommendations—A Narrative Review

**DOI:** 10.3390/healthcare13172222

**Published:** 2025-09-04

**Authors:** Sebastian Żur, Adam Sokal, Wiktoria Staśkiewicz-Bartecka, Agata Kiciak, Mateusz Grajek, Karolina Krupa-Kotara, Oskar Kowalski, Agnieszka Białek-Dratwa

**Affiliations:** 1 Department of Human Nutrition, Department of Dietetics, Faculty of Public Health in Bytom, Medical University of Silesia in Katowice, Jordana 19, 41-808 Zabrze, Poland; 2 Department of Food Technology and Quality Evaluation, Department of Dietetics, Faculty of Public Health in Bytom, Medical University of Silesia in Katowice, Jordana 19, 41-808 Zabrze, Poland; 3 Department of Public Health, Faculty of Public Health in Bytom, Silesian Medical University in Katowice, ul. Piekarska 18, 41-902 Bytom, Poland; 4 Department of Epidemiology, Department of Epidemiology and Biostatistics, Faculty of Public Health in Bytom, Silesian Medical University in Katowice, ul. Piekarska 18, 41-902 Bytom, Poland

**Keywords:** Down syndrome, children, diet, nutritional recommendations, feeding disorders, food neophobia

## Abstract

**Background/Objectives:** Children with Down syndrome (DS) present unique and multifaceted nutritional challenges arising from genetic, metabolic, and developmental factors. Despite growing interest in the health of individuals with DS, dedicated nutritional guidelines tailored to their specific needs remain lacking. This narrative review aims to summarize current scientific evidence on nutritional status, challenges, and therapeutic strategies in children with DS, with an emphasis on clinical implications and practical recommendations for healthcare professionals. **Methods:** A literature search was conducted across four databases (PubMed, Scopus, Web of Science, and Google Scholar) for English-language publications from 1993 to June 2025. Thirty-five peer-reviewed articles were included, comprising original studies, narrative reviews, and expert guidelines (e.g., the American Academy of Pediatrics [AAP], the European Society for Paediatric Gastroenterology, Hepatology and Nutrition [ESPGHAN], and the European Federation of Associations of Dietitians [EFAD]). The selection process followed the PRISMA protocol. Studies were categorized according to key themes: energy requirements, comorbidities, feeding difficulties, nutrient needs, and therapeutic interventions. **Results:** Children with DS typically exhibit lower basal metabolic rates and altered body composition (i.e., higher fat mass and reduced lean mass), which increase their risk of both obesity and nutrient deficiencies. Common comorbidities—such as hypothyroidism, celiac disease, and gastrointestinal or immune disorders—further complicate dietary management. Feeding difficulties, including sucking/swallowing impairments, food selectivity, neophobia, and delayed independence in eating, are prevalent and significantly affect diet quality. Key nutrients of concern include protein, omega-3 fatty acids, fiber, vitamins B12 and D, iron, and antioxidants. Although no official nutrition guidelines currently exist for this population, existing recommendations from pediatric and dietetic organizations provide partial guidance that can be adapted to clinical practice. **Conclusions:** There is an urgent need to develop evidence-based, population-specific dietary guidelines for children with Down syndrome. Clinical nutrition care should be individualized, multidisciplinary, and proactive, integrating regular assessments of growth, feeding abilities, and biochemical markers. Dietitians must play a central role in both early intervention and long-term management. Further research, particularly interventional studies, is essential to optimize dietary strategies and improve health outcomes in this vulnerable population.

## 1. Introduction

Down syndrome (DS), also known as trisomy 21, is the most common chromosomal abnormality in humans, caused by the presence of an additional copy of chromosome 21. Its incidence is estimated at approximately 1 in 700 live births. The risk of having a child with DS increases significantly with maternal age; in women over 40 years, it can be as high as 1 in 100 births [1]. Globally, it is estimated that 5–6 million people are currently living with DS. Advances in medical care, including improved management of congenital anomalies and enhanced healthcare, have contributed to a marked increase in life expectancy in this population, which now exceeds 60 years in developed countries [2].

DS is associated with numerous clinical features, including cognitive impairment, delays in physical and motor development, and a wide range of comorbidities. The most common health problems include congenital heart defects (present in 40–60% of patients), muscle hypotonia, hearing and vision impairments, hypothyroidism (15–20%), gastroesophageal reflux disease, chronic constipation, celiac disease (5–12%), and immune dysfunction [3,4,5]. These conditions have a substantial impact on the nutritional status of children with DS. Epidemiological studies indicate that this population is particularly vulnerable to both nutrient deficiencies and excess body weight. Overweight and obesity are reported in 23–62.5% of children and adolescents with DS [6]. At the same time, children with DS frequently present with specific feeding and eating difficulties, such as food selectivity, chewing and swallowing impairments, and delayed acquisition of independent eating skills [7].

Moreover, reduced physical activity, in combination with these challenges, further increases the risk of metabolic disorders [8]. Despite the growing body of research on the medical and developmental aspects of DS, nutritional guidelines for this population remain limited. In most cases, standard pediatric dietary guidelines are applied without considering the unique needs of children with DS. Given the rising life expectancy and the critical role of early dietary intervention, there is an urgent need to develop and implement tailored nutritional recommendations for this group.

The objective of this narrative review is to critically synthesize the current scientific evidence on the nutritional requirements of children with DS and to evaluate existing clinical guidelines and evidence-based recommendations. The review addresses the most common nutritional challenges observed in this population, the importance of specific nutrients, the potential role of dietary supplementation, meal composition strategies, and current positions advocated by leading scientific and professional organizations.

## 2. Materials and Methods

### 2.1. Search Strategy

The literature search was conducted using four databases: PubMed, Scopus, Web of Science, and Google Scholar. Publications available up to 30 June 2025, were considered for inclusion. The following keyword combinations were applied: “Down syndrome,” “children,” “diet,” “nutritional recommendations,” “feeding disorders,” “feeding problems,” and “food neophobia.” The time frame of 1993–2025 was selected due to the limited number of articles addressing nutritional issues in children with DS. In total, thirty-five English-language articles were included in the final analysis.

### 2.2. Inclusion and Exclusion Criteria

The review included original research articles, narrative reviews, and position papers issued by scientific organizations (e.g., the American Academy of Pediatrics [AAP], the European Society for Paediatric Gastroenterology, Hepatology and Nutrition [ESPGHAN], and the European Federation of Associations of Dietitians [EFAD]). Eligible publications addressed children with Down syndrome and, as supplementary material, adolescents. Studies considered relevant covered aspects of nutrition, motor and psychophysical development, nutritional status, feeding disorders, food preferences, and diet therapy, and were available in English.

**Figure 1:**
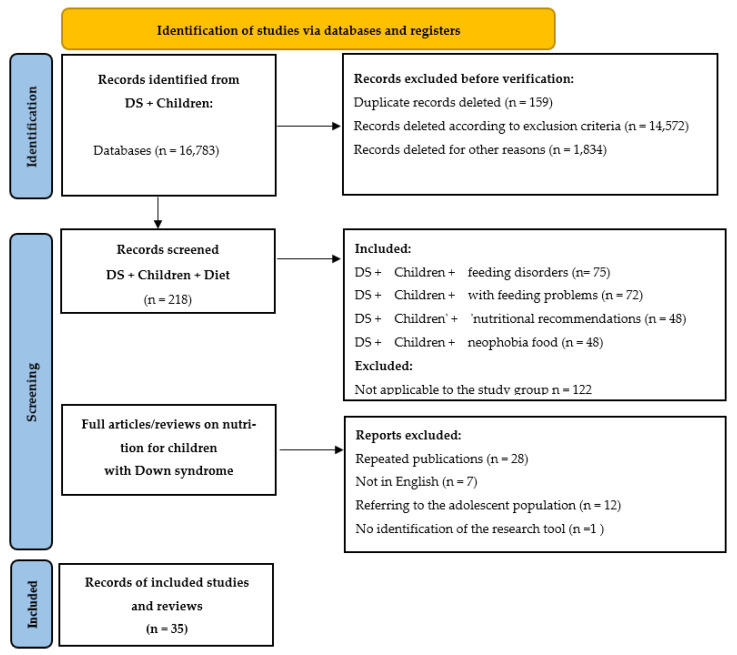
PRISMA diagram showing the process of identification, selection, eligibility assessment, and inclusion of articles in this review.

The following were excluded from the review: non–peer-reviewed articles, case reports, publications lacking nutritional content or unrelated to dietary practice, and studies focused exclusively on adults with DS (unless findings could reasonably be generalized to children). The selection process was documented using the PRISMA flow diagram (Figure 1), which outlines the stages of identification, screening, eligibility assessment, and final inclusion of articles. To ensure the most comprehensive coverage possible, database searches were performed separately in PubMed, Scopus, Web of Science, and Google Scholar [9,10]. A total of thirty-five English-language articles met the inclusion criteria and were analyzed.

### 2.3. Data Synthesis

After duplicate removal, two authors independently screened the abstracts and full texts of the publications for relevance to the topic and clinical applicability. In cases of disagreement, decisions were resolved through consensus. The extracted data were organized into the following thematic categories: (1) body composition and energy requirements, (2) comorbidities and diet, (3) feeding disorders and sensory preferences, and (4) nutritional and therapeutic recommendations. In addition, data from studies conducted in Polish populations of children with DS were included, as they may provide valuable context-specific information.

Given the limited number of interventional studies involving children with DS, this review primarily draws upon observational and retrospective data, expert opinions, and position statements from scientific organizations. A narrative review method was employed, which—despite its broad analytical scope—does not allow for a formal assessment of the quality of evidence according to the evidence-based medicine (EBM) hierarchy.

Figure 1 presents the PRISMA flow diagram illustrating the inclusion and exclusion process for articles in this review. Independent searches were conducted in PubMed, Scopus, Web of Science, and Google Scholar [9,10]. In total, the review included 30 studies addressing nutrition, feeding, oromotor function, and health status in children with DS: 2 publications containing recommendations, 10 narrative reviews, 5 other studies, and 13 original research articles.

Table 1 presents the structure of the research question, developed using the PICO model (Population, Intervention, Comparison, Outcome), a framework commonly applied in systematic reviews and consistent with PRISMA guidelines [9,10]. The PICO framework was employed to formulate the research question, define inclusion and exclusion criteria, and guide the search strategy. This approach ensured consistency in study selection and data extraction, while enhancing the transparency and clinical relevance of the review. Table 1 provides a detailed description of the study population, the types of nutritional interventions analyzed, potential comparison groups, and the expected outcomes of dietary strategies in children with Down syndrome. Table 2 presents a systematic summary of the available literature on nutrition, feeding behaviors, oromotor function, motor skills, and selected aspects of health in children and adolescents with Down syndrome. This table includes original research articles, narrative reviews, and recommendation papers, outlining study populations, research objectives, methods and assessment tools, as well as the key findings.

## 3. Results

### 3.1. Characteristics of the Nutritional Needs of Children with Down Syndrome

Children with DS have specific nutritional needs arising from genetic factors, physiological abnormalities, and associated comorbidities. Consequently, standard nutritional guidelines developed for neurotypical children may be insufficient for this population. It is essential to individualize dietary recommendations by considering the child’s overall health status, lifestyle, level of physical activity, and cognitive abilities, as well as the capabilities and circumstances of their family [3,4,5,6,7,8].

#### 3.1.1. Reduced Energy Requirements in Children with Down Syndrome

One of the most critical aspects of nutritional planning for children with DS is their reduced basal metabolic rate (BMR). Studies have shown that total energy expenditure in children with DS may be, on average, 10–15% lower than in their healthy peers, which constitutes a significant predisposing factor for excessive weight gain [11]. The increased susceptibility of children with DS to overweight and obesity is associated with reduced lean body mass, muscle hypotonia, and limited physical activity. Consequently, weight gain may occur even with a moderate energy intake [6].

#### 3.1.2. Body Composition of Children with Down Syndrome

Body composition analyses have demonstrated that children and adolescents with DS have significantly lower lean body mass and a higher percentage of body fat compared with peers without trisomy 21, despite comparable body mass index (BMI) values [22]. These discrepancies may lead to the misclassification of nutritional status if assessment relies solely on BMI. Therefore, in evaluating the nutritional status of children with Down syndrome, it is particularly recommended to use body composition analyzers (e.g., bioelectrical impedance analysis [BIA]) and to perform regular measurements of skinfold thickness and body circumferences. Alternatively, DS-specific growth and centile charts can be applied [23].

#### 3.1.3. Comorbidities and Nutritional Needs of Children with Down Syndrome

Coexisting endocrine, gastroenterological, and autoimmune disorders have a major influence on nutrient requirements and tolerance:

- **Hypothyroidism**—estimated to occur in over 15% of children with Down syndrome, it affects the metabolic rate and may increase weight gain [4]. It requires strict control of the energy content of the diet and the intake of iodine, selenium, and iron.
- **Celiac disease**—diagnosed in 4.6–7.1% of children with DS [4,12,13]. If coexisting, a gluten-free diet is necessary, as it is the only effective treatment for this condition. Due to the restriction of gluten-containing cereal products, an elimination diet may lead to deficiencies in dietary fiber, B vitamins (especially B1, B6, and folic acid), and iron, which justifies the need for monitoring and appropriate supplementation [24].
- **Gastroenterological disorders** are common clinical problems observed in children with Down syndrome. The most common disorders include gastroesophageal reflux and chronic constipation, which may be functional or result from coexisting anatomical anomalies of the gastrointestinal tract. Due to the diverse etiology of symptoms, an individual diagnostic and therapeutic approach is necessary in treatment [25].
- **Immune system disorders**, which are often observed in children with Down syndrome, predispose them to recurrent infections, which can lead to periodic loss of appetite and require temporary modification of the structure and nutritional value of the diet [26].

#### 3.1.4. Physical Activity in Children with Down Syndrome

Motor development limitations and reduced muscle tone, characteristic of children with DS, contribute to decreased spontaneous physical activity and increased sedentary time. Compared with peers without trisomy 21, children with DS demonstrate significantly lower levels of spontaneous activity, which may hinder achievement of the WHO-recommended 60 min of moderate-to-vigorous physical activity per day. Given the elevated risk of insulin resistance and predisposition to metabolic syndrome in this population, both total dietary energy intake and macronutrient distribution should be individually tailored to the patient’s metabolic status, taking into account physical activity level, body composition, and biochemical parameters [6,8,27].

### 3.2. Nutritional Disorders Typical of Children with Down Syndrome

Eating disorders in children with DS are complex and multifactorial, arising from anatomical, neurological, and behavioral factors. They occur far more frequently than in the general population and have a profound impact on nutritional status, growth, and psychophysical development.

#### Sucking, Swallowing, and Biting Disorders

From the earliest stages of life, and for several months after birth, children with DS may experience difficulties with sucking, swallowing, and chewing. These disorders are primarily attributable to reduced muscle tone (hypotonia), particularly in the oral and facial muscles; a short hard palate and high palatal arch, which impair swallowing coordination; an enlarged tongue (macroglossia), often protruding beyond the oral cavity; and impaired coordination of sucking, swallowing, and breathing, which is especially critical in infancy [14,28]. During feeding, children with DS frequently present with masticatory dysfunction and aspiration episodes, both of which can significantly hinder the timely and appropriate diversification of the diet [29,30].

The literature to date has addressed sucking and swallowing disorders in infants with DS only to a limited extent. Findings from studies conducted in clinical populations, including both infants and adults, indicate that up to 80% of individuals with DS experience difficulties related to food intake. However, these results should be interpreted with caution, as the analyses were based on clinical groups, which limits their generalizability to the broader DS population [15]. Caregivers of children with DS often report a need for feeding support, yet may fail to recognize or may underestimate their child’s difficulties with eating. Therefore, it is recommended that meals be consumed under adult supervision to allow for the early detection of feeding disorders and to ensure nutritional safety [29].

### 3.3. Selectivity and Food Neophobia

Another critical issue is difficulty in accepting new foods and textures. Food selectivity in children with DS may be related to limited sensory processing (e.g., hypersensitivity to texture, temperature, or taste), fear of choking due to previous experiences or anatomical swallowing difficulties, as well as routine and the need for predictability (a common characteristic in children with intellectual disabilities) [16,31]. Children may prefer a limited range of products (e.g., yogurt, bread, bananas) while rejecting entire food groups (e.g., vegetables or meat). Such dietary patterns can result in deficiencies of vitamins, minerals, and dietary fiber.

Food selectivity—defined as limited acceptance of a variety of foods—and food neophobia—the reluctance or fear of trying new foods—are common in the pediatric population, and their severity may increase with age, particularly in the absence of appropriate therapeutic interventions [31]. Although precise data on the prevalence of these disorders in children with DS are lacking, oral-motor deficits and sensory preferences, especially regarding texture, are thought to contribute to dietary restrictions. A study by Ross et al. reported that children with DS were more likely to accept foods with soft, smooth, soluble, or puréed textures, while they experienced difficulties with foods requiring intensive chewing or those with hard or sticky consistencies [17]. Similarly, Hopman et al. found that children with DS exhibited more frequent problems with food consistency due to delayed oral-motor development compared with typically developing peers [32]. Such sensory-driven preferences may indirectly restrict dietary variety, increasing the risk of nutritional deficiencies and negatively affecting the development of chewing and speech functions.

### 3.4. Delayed Development of Independent Eating

Excessive appetite and disturbances in hunger and satiety signals—currently, there is no conclusive scientific research confirming the occurrence of hyperphagia and disturbances in satiety regulation mechanisms as typical features in the population of children with Down syndrome. Nevertheless, parental reports and clinical observations sometimes highlight a tendency in some children with DS to eat despite not feeling hungry, difficulty in recognizing satiety signals, and a tendency to snack frequently. Although these are anecdotal data, they may suggest the existence of individual differences in appetite neuroregulation in some children with Down syndrome. Further scientific research is needed to understand better the physiological mechanisms underlying appetite regulation in this group. Considering all of the above, it is necessary to tailor therapeutic interventions to the specific needs of children with Down syndrome, taking into account their developmental difficulties [6,18,33,34,35]. The development of independent eating skills—such as using cutlery, drinking from an open cup, or participating in family meals—is slower in children with DS compared with the general population. These delays arise from difficulties in several domains:

- Fine motor skills (hand and finger coordination). Studies indicate that children with DS demonstrate delays in motor development, which affect their ability to manipulate objects such as cutlery and cups. For example, analyses of motor test results have shown significant impairments in both fine and gross motor skills [18].
- Visual-motor perception. Virji-Babul et al. reported that children with DS have difficulties in recognizing biological motion, suggesting impaired processing of global movement patterns. These deficits may adversely affect visual-motor integration, which is crucial for activities such as independent eating. The authors highlight the importance of early interventions aimed at enhancing visual integration to support skill acquisition in this group [19].
- Motor planning (praxis). A study by Fidler and colleagues (2005) assessed praxis skills in young children with DS, children with other developmental disabilities, and typically developing peers. Findings demonstrated significant deficits in planning and executing purposeful, sequential motor actions among children with DS. Notably, difficulties were observed in tasks requiring gesture imitation and performance of movement commands based on verbal instructions. The authors emphasize that praxis impairments can substantially limit the acquisition and automation of complex everyday skills—such as eating, dressing, and hygiene—underscoring the need for early, targeted therapy to support functional development in this population [20].

Considering these factors, therapeutic interventions should be tailored to the specific developmental challenges of children with Down syndrome, with particular attention to individualized support for motor, perceptual, and regulatory domains [6,33,34,35].

### 3.5. Key Nutrients in the Diet of Children with Down Syndrome

The diet of children with DS should be complete, diverse, and individually tailored. Given their specific metabolic predispositions, health burdens, and feeding disorders, particular attention should be directed to the intake of selected nutrients. The following nutrients are those most commonly found to be deficient or excessive in children with DS and may significantly influence somatic growth, nervous system function, immune competence, and the prevention of chronic diseases [21].

#### 3.5.1. Protein

Protein plays a fundamental role in child development, contributing to growth, tissue regeneration, and the proper functioning of the immune system. In the population of children with Down syndrome, Nordstrøm et al. emphasized the need for detailed nutritional assessments, particularly in underweight patients and those with clinical signs of feeding difficulties. The authors also highlighted the importance of early dietary intervention, safe feeding practices, and the development of food acceptance skills. Although the study did not directly specify protein sources, it underscored the necessity of individualized dietary recommendations, especially during periods of rapid growth or in the presence of inflammatory or autoimmune conditions [36].

Protein intake should be diversified, incorporating both plant- and animal-based sources. Increased protein requirements must be considered in malnourished as well as overweight individuals [5]. According to healthy eating principles, each meal should include a source of protein, ideally of animal origin. Meat is also a key dietary source of vitamin B12, the deficiency of which may result in macrocytic anemia, among other complications. A study by Białek-Dratwa et al. demonstrated that although almost all parents reported including meat in their children’s diets, only 48.72% provided it with sufficient frequency [21].

#### 3.5.2. Fats

Although explicit confirmation is lacking in the scientific literature, it is widely believed that children with DS may present with lipid metabolism abnormalities, including elevated LDL cholesterol and triglyceride levels, already in preschool age. Omega-3 fatty acids—particularly eicosapentaenoic acid (EPA) and docosahexaenoic acid (DHA)—are considered essential, as they are a critical component of diets supporting neurological development and immune function in children with Down syndrome. DHA is a major structural constituent of the brain and retina, and adequate intake may promote cognitive development, visual function, and modulation of inflammatory processes, which may be exacerbated in children with DS. Moreover, both EPA and DHA exert anti-inflammatory and cardioprotective effects, which are particularly relevant given the increased risk of cardiovascular disease and immune dysregulation observed in this population. Although well-designed interventional studies in children with DS are still required, current evidence suggests that including dietary sources of long-chain omega-3 fatty acids—such as fatty marine fish, algal oils, or supplements—may provide health benefits, especially during periods of rapid neurodevelopment and in the presence of inflammatory or autoimmune conditions [37].

Polish researchers have suggested that fat intake in individuals with DS may need to be reduced compared with standard dietary recommendations, particularly those applied in liver and pancreatic diseases. Given the elevated cardiovascular risk and the presence of oxidative stress, dietary strategies should emphasize reducing saturated fatty acid intake while ensuring adequate consumption of monounsaturated and polyunsaturated fatty acids, with particular focus on omega-3 polyunsaturated fatty acids [5].

#### 3.5.3. Carbohydrates

The primary source of carbohydrates in the diet of individuals with DS should be complex carbohydrates, predominantly provided by vegetables, fruits, whole-grain products, and legumes. Such a dietary pattern supports healthy weight maintenance and reduces the risk of metabolic disorders. The type and amount of dietary fiber may require adjustment in cases of gastrointestinal disease, such as inflammation of the intestines, stomach, liver, or pancreas [5]. As in the general population, limiting added sugar intake is essential to avoid exceeding recommended levels, and total carbohydrate intake should be carefully controlled in individuals who are overweight, prediabetic, or diabetic [18]. Notably, a study by Kmiecik et al. reported that 91% of Polish respondents admitted to purchasing sweets, with the majority considering them an essential part of their diet [38].

### 3.6. Characteristics of Nutritional Challenges in Children with Down Syndrome

Children with DS require a tailored nutritional approach due to coexisting metabolic and endocrine disorders as well as specific somatic and neurodevelopmental characteristics. They demonstrate an increased susceptibility to overweight and obesity, resulting not only from reduced basal metabolic rate but also from low physical activity, muscle hypotonia, and often inadequate eating habits shaped within the home environment [36].

In the Polish population, a cross-sectional study conducted among 195 parents of children and young adults with DS showed that 20.9% of participants were overweight (15.3% overweight and 5.6% obese). Strikingly, 94.4% of the children were not included in any dietary program. These findings underscore the lack of systemic specialist support and the potential risk of further nutritional errors due to insufficient knowledge among parents and caregivers [21]. The same study also reported very low fruit and vegetable consumption, with most children consuming them only once daily: vegetables in 42.6% and fruit in 44.6% of respondents [21].

Such insufficient intake of fruits and vegetables—key sources of fiber, antioxidant vitamins, and minerals—may contribute to nutritional deficiencies and exacerbate pro-inflammatory processes, which are already heightened in children with DS due to reduced immunity and frequent comorbidities [19]. In addition, inappropriate nutrient elimination without medical justification was common. For example, 28.7% of children had lactose removed from their diets, although only 9.7% had confirmed intolerance—indicating that just 33.9% of such exclusions were medically warranted. In the long term, these practices may contribute to secondary intolerances, nutritional deficiencies, and gut microbiota imbalances [21].

In light of these findings, it can be clearly concluded that children with DS represent a population at increased risk of nutritional errors that may adversely affect both somatic development and the course of comorbidities. Therefore, it is essential to develop precise, evidence-based dietary guidelines tailored to the needs of this group and to implement an interdisciplinary model of care in which clinical dietitians play a central role.

### 3.7. Diet Therapy Models and Current Guidelines

Despite the growing body of research on the health and developmental aspects of children with Down syndrome, no consistent, comprehensive, and official dietary guidelines have yet been established for this population. In clinical practice, standard nutritional recommendations for healthy children are typically adapted to address individual needs arising from comorbidities. Nonetheless, several documents and models of practice can serve as valuable reference points for dietitians and multidisciplinary therapeutic teams.

#### 3.7.1. Recommendations of the American Academy of Pediatrics (AAP):

- The most comprehensive document on the care of children with DS is the American Academy of Pediatrics (AAP) position statement “Health Supervision for Children and Adolescents With Down Syndrome” [3].
- Although the document does not include a dedicated chapter on nutritional recommendations, it addresses several aspects relevant from a clinical nutrition perspective. These include the following:
  1. growth monitoring using centile charts specifically developed for children with Down syndrome;
  2. regular assessment of biochemical parameters, including iron, ferritin, vitamin D, TSH, and glucose levels;
  3. promotion of physical activity and a healthy lifestyle;
  4. weight control and early nutritional intervention in cases of risk for overweight or malnutrition.
- The AAP also emphasizes the importance of an interdisciplinary approach and recommends ensuring access to a clinical dietitian for children with DS in cases of feeding disorders, gastrointestinal diseases, abnormal weight gain, or the need for an elimination diet [3]

#### 3.7.2. ESPGHAN and EFAD Guidelines

The European Society for Paediatric Gastroenterology, Hepatology and Nutrition (ESPGHAN) and the European Federation of Associations of Dietitians (EFAD) have not yet published dedicated nutritional guidelines for children with Down syndrome. However, the 2017 ESPGHAN guidelines on the nutritional management of children with neurological disorders highlight the importance of individualizing dietary recommendations for children with intellectual disabilities and functional limitations [39]. The document recommends, among other things:

- reducing energy intake in children with limited physical activity and delayed development, while increasing the nutritional density of the diet;
- monitoring growth and adjusting food consistency according to oromotor abilities;
- implementing nutritional interventions based on a comprehensive assessment of nutritional status, including laboratory tests and evaluation of eating habits;
- ensuring interdisciplinary care involving physicians, dietitians, feeding therapists, physiotherapists, and psychologists.

Although these guidelines are not specifically targeted at children with Down syndrome, they may be successfully applied to this population given the similar functional and nutritional profiles observed in many patients [39].

## 4. Summary and Practical Recommendations

Children with DS represent a group of patients with unique and multidimensional nutritional needs. Owing to the coexistence of genetic, physiological, developmental, and environmental factors, they require a personalized dietary approach that is holistic, interdisciplinary, and evidence-based. Below is a summary of the most critical findings from the literature review, followed by specific practical recommendations for implementation in routine dietary care.

### 4.1. Conclusions from the Literature Review

1. **Growth disorders and body composition abnormalities.** Children with Down syndrome are typically characterized by shorter stature, higher body fat levels, and reduced lean body mass. Their basal metabolic rate (BMR) is significantly lower, which increases the risk of overweight and obesity, even with an apparently “normal” energy intake.
2. **High prevalence of comorbidities.** Conditions such as hypothyroidism, celiac disease, constipation, gastroesophageal reflux, and immune disorders substantially affect nutrient tolerance and absorption, necessitating the continuous monitoring of nutritional status and biochemical parameters.
3. **Feeding difficulties and food selectivity.** Delayed development of oromotor functions, limited acceptance of textures, sensory preferences, and challenges with eating independence are common and have a critical impact on diet quality. Without appropriate intervention, these issues may result in nutrient deficiencies and contribute to social isolation.
4. **Lack of official, dedicated guidelines.** To date, no country has developed comprehensive dietary recommendations that take into account the specific needs of children with Down syndrome, including those related to feeding disorders. In clinical practice, general pediatric guidelines are typically applied and subsequently adapted to the particular requirements arising from developmental disabilities and comorbidities, with a regular assessment of individual patient needs. It should be emphasized that although some organizations (e.g., AAP, ESPGHAN) provide partial recommendations relevant to this population, they do not constitute dedicated, comprehensive nutritional guidelines for children with Down syndrome.

### 4.2. Practical Recommendations for Therapeutic Teams

Based on the review of the available scientific evidence, the following recommendations have been developed to provide a practical guide for dietitians, physicians, therapists, other healthcare professionals, and families. To enhance transparency, recommendations supported by scientific evidence are labeled as *Evidence-Based Medicine* (EBM), whereas those derived primarily from expert consensus and clinical practice are labeled as *Clinical Practice* (CP).

#### 4.2.1. Assessment and Monitoring of Nutritional Status

- Use dedicated centile charts for children with DS (e.g., growth charts for children with Down syndrome) (EBM).
- Measure height, weight, BMI, waist circumference, body composition (BIA), and skinfold thickness every 6–12 months (EBM).
- Conduct a nutritional interview, taking into account meal patterns, consistency, preferences, selectivity, and the presence of eating difficulties (EBM).

#### 4.2.2. Diet

- Meals should be regular (4–5 per day), calorie-controlled (10–15% reduction from normal intake), and rich in nutrients (EBM).
- Include good sources of protein, healthy fats (including omega-3), and complex carbohydrates (EBM).
- Adjust the consistency to the child’s oral motor skills, but avoid prolonged use of purees or blended foods (EBM).

#### 4.2.3. Nutritional Therapy to Support Independence

- Work with a speech therapist/neurological speech therapist and feeding therapist to improve oromotor function (EBM).
- Encourage your child to participate in meal preparation and make simple decisions (“What to eat?”) (CP).
- Use adaptive aids: spoons, forks, plates with rims, cups with handles (CP).
- Educate the family on positive modeling and eliminating pressure during mealtimes (CP).

#### 4.2.4. Interdisciplinary Cooperation

- Set up a therapeutic team (doctor, clinical dietitian, speech therapist/neurological speech therapist, psychologist, physical therapist) and schedule regular meetings every 6 months (CP).
- Document progress, modify recommendations on an ongoing basis, and adapt them to the child’s changing needs (CP).

#### 4.2.5. Future Needs—Research Perspective

Due to the increasing average life expectancy of people with Down syndrome, as well as improvements in their quality of life and medical care, it is crucial to apply the following:

- develop official population-based nutritional guidelines for children and adults with DS(CP).
- conduct randomized clinical trials on the effectiveness of dietary and supplementation interventions in this group (EBM),
- implement nationwide educational and preventive programs aimed at families, schools, and institutions supporting people with intellectual disabilities (CP).

### 4.3. Summary

DS is a multidimensional genetic disorder whose clinical and developmental course necessitates an individualized nutritional approach. A review of the literature demonstrates that children with DS are characterized by reduced basal energy expenditure, a higher proportion of body fat combined with decreased lean body mass, limited physical activity, and a high prevalence of comorbidities (including hypothyroidism, celiac disease, constipation, and immune disorders). These factors contribute to a dual risk: malnutrition on the one hand, and overweight and obesity on the other. In addition, feeding difficulties—such as problems with sucking, chewing, and swallowing, as well as picky eating, food neophobia, and delayed development of mealtime independence—significantly impair diet quality and increase the risk of nutritional deficiencies.

The absence of dedicated nutritional guidelines for children with DS at both international and national levels means that dietary care relies largely on the adaptation of general pediatric recommendations, which often prove insufficient given the specific metabolic and functional needs of this population. Although some organizations, such as the American Academy of Pediatrics (AAP), have attempted to systematize care for children with Down syndrome, nutritional aspects remain marginal or fragmented. Similarly, the positions of ESPGHAN and EFAD, while not directly focused on DS, provide important recommendations for children with intellectual disabilities that may be applied to this group.

The available scientific evidence underscores the need to implement a holistic, interdisciplinary model of care in which clinical dietitians play a central role in both prevention and dietary management of comorbidities. Recommended strategies should include systematic monitoring of growth and body composition (preferably with bioelectrical impedance analysis and DS-specific centile charts), individualized adjustment of energy intake and food texture, modification of macronutrient distribution according to clinical status, and the implementation of feeding therapy to support the development of oromotor functions and independent eating skills.

### 4.4. Conclusions for the Future

1. **Need to develop dedicated nutritional guidelines.** There is a clear gap in official, evidence-based dietary recommendations for children with Down syndrome. It is essential to establish expert working groups within national and international organizations (e.g., EFAD, ESPGHAN, PTD) to develop comprehensive, population-specific guidelines tailored to the unique needs of this group.
2. **Development of intervention studies.** The vast majority of data on nutrition in children with DS are derived from observational research. There is an urgent need for randomized clinical trials to evaluate the effectiveness of specific dietary interventions—such as supplementation strategies, family education programs, and feeding therapy—in improving nutritional status, psychomotor development, and quality of life.
3. **Inclusion of nutrition in early intervention programs.** Incorporating nutritional assessment and care into early intervention services, along with the inclusion of clinical dietitians in multidisciplinary developmental support teams, would enable the early identification and management of feeding disorders in infancy, which may be critical for preventing somatic and functional developmental abnormalities.
4. **Integration of nutritional care into healthcare and education systems.** Developing and implementing structured dietary care models integrated into educational institutions, daycare facilities, and rehabilitation centers is crucial. Training for parents, teachers, and therapists should systematically include a nutritional component.
5. **Expansion of preventive and educational measures.** In light of the increasing life expectancy and improved medical care of individuals with DS, it is necessary to implement nationwide nutritional prevention programs that encompass children, adolescents, and adults with Down syndrome.

## 5. Strengths and Limitations of the Review

The presented review is narrative in nature, which entails certain methodological limitations. Due to the narrative nature of this publication, no formal assessment of the methodological quality of the included studies was performed using standardized tools. The lack of such an analysis limits the possibility of unambiguously determining the reliability of individual results. It reduces the strength of the conclusions, which should be taken into account when interpreting the recommendations and clinical implications presented. The included publications are characterized by considerable heterogeneity in terms of research methods, age, and size of the groups of children, adolescents and young adults with DS, as well as the diagnostic tools used, which limits the possibility of the direct comparison of results and the formulation of generalized recommendations. The number of studies on nutrition in children with Down syndrome is minimal, and the available studies are primarily observational or retrospective, with small sample sizes and no randomization. Many of the studies included relatively small sample sizes, often limited to a dozen or so participants, which significantly reduces the statistical power of the analyses and limits the possibility of generalizing the results to the broader population with DS. At the same time, the authors of this manuscript conducted the two most extensive studies included in this review. On the one hand, this is a valuable source of data on the nutrition of children and adolescents with DS. Still, this fact may generate a risk of potential interpretative bias, which should be taken into account when assessing the reliability and generalizability of the conclusions of this narrative review.

However, this review is one of the few attempts at a comprehensive analysis of nutritional issues in the population of children with DS. Its strength lies in its broad scope, covering both metabolic and clinical aspects, as well as issues related to feeding, food selectivity, oromotor function and the development of independence in eating. As a result, this publication is holistic in nature, highlighting the multidimensional nature of the nutritional needs of this pediatric population.

The practical value of this work also lies in the formulation of recommendations for therapeutic teams, which can be directly applied in everyday clinical practice. At the same time, the review highlights the existing gap in the form of a lack of official, dedicated nutritional guidelines for children with DS. It thus points to the need to develop dietary recommendations and policies based on the results of intervention studies and expert consensus in the future, which is an essential direction for further scientific and clinical work.

Despite the methodological limitations taken into account, this review makes an essential contribution to the development of clinical practice in the nutrition of children and adolescents with DS, including a synthesis of available data on nutrition and feeding difficulties in DS, practical recommendations, and a clear indication of the need for further intervention studies and the development of official nutritional guidelines for this population.

## Figures and Tables

**Table 1:** Structure of the research question according to the PICO scheme.

Element	Detailed Description	Example Wording
**P (Population)**	Children and adolescents with DS (aged 0–18), regardless of gender, degree of intellectual disability or comorbidities.	*Children and adolescents aged 0–18 years diagnosed with DS (Trisomy 21).*
**I (Intervention)**	All dietary and nutritional interventions, such as individual diet therapy, nutrition education for parents, supplementation (e.g., DHA, vitamin D), modification of food texture, elimination diets (e.g., gluten-free), and programs to support independent eating.	*Nutritional interventions including dietary counseling, food texture modification, supplementation, or elimination diets.*
**C (Comparison)**	No intervention or standard dietary management used in children without Down syndrome; possibly comparison of different dietary strategies (e.g., intervention A vs. B).	*Standard dietary care or no nutritional intervention.*
**O (Outcomes)**	Changes in nutritional status (body weight, BMI, body composition), eating behaviors (food selectivity, independence), oromotor functions, quality of life, reduction in dietary errors.	*Improvement in nutritional status, reduction in obesity risk, increased dietary diversity, improved self-feeding abilities.*

**Table 2:** Characteristics of studies on nutrition, feeding, oromotor functions, and health status of children with DS—literature review.

Author, Year	Research Group (n, Age)	Research Objective/Area	Tools/Methods Used	Key Findings
Anil, M.A., Shabnam, S., & Narayanan, S. (2019). [7]	17 children with DS (10 girls and 7 boys) and 47 typically developing children (20 girls and 27 boys).	Assessment of possible feeding and swallowing problems in children with DS aged 2–7 years	• Questionnaire for assessing feeding problems in children with DS (developed and validated) • Com-DEALL Checklist (assessment of oromotor skills) • Feeding Handicap Index for Children—FHI-C (assessment of the impact of problems) • Video recordings of feeding sessions • Direct observation of feeding with different foods	• Children with DS are more likely to have feeding problems—mainly with food consistency, swallowing and control in the mouth. • Reduced oromotor abilities—weaker movements of the jaw, tongue and lips compared to the control group. • Greater burden on families—parents reported significantly more difficulties in all domains (physical, functional, emotional).
Xanthopoulos, M.S., Walega, R., Xiao, R., Pipan, M.E., Cochrane, C.I., Zemel, B.S., Kelly, A., & Magge, S.N. (2022) [8]	77 young people with DS (aged 10–20; and 57 people without DS, matched for age, gender, race, ethnicity and BMI	Assessment of physical activity levels and their association with visceral adiposity (VFAT) in adolescents with Down syndrome	• Sense Wear Mini accelerometer—7-day activity recording (min/day LPA, MVPA, SA, steps) • Anthropometry (BMI-Z, height, weight) • Body composition—DXA (visceral fat, VFAT) • Assessment of puberty according to stages	• Adolescents with DS took fewer steps/day and performed less MVPA (trend), but spent more time in LPA. • 63% of DS met the recommendations of ≥60 min MVPA/day (no difference vs. control group). • LPA inversely and SA positively correlated with BMI-Z and VFAT. • MVPA was negatively associated with BMI-Z and VFAT, especially in boys.
Luke, A., Sutton, M., Schoeller, D.A., Roizen, N.J.M. (1996) [11]	10 children with DS (aged 5–11) and 10 healthy children	Assessment of body composition, energy expenditure, and energy and nutrient intake in children with DS compared to a control group	• Deuterium dilution (TBW, FFM) • Electrical bioimpedance (BIA) • Skin fold measurement (4-point) • Double-labeled water (energy expenditure) • 3-day food intake records (1 weekend day, 2 weekdays) • Food Processor II diet analysis	• 50% of children with DS met the criteria for obesity (weight/height index > 1.2). • No differences in body composition were found between the groups (FFM, FM). • Energy intake was significantly lower in the DS group in relation to the RDA. • Lower intake of riboflavin, vitamin B6, iron, and calcium in DS vs. control. • 50% of children with DS had Ca/Zn deficiencies; 80% had low copper intake.
Carnicer, J., Farre, C., Varea, V., Vilar, P., Moreno, J., Artigas, J. (2001) [12]	284 people with DS (aged 1–25), Spain	Assessment of the prevalence of coeliac disease in individuals with DS	• Determination of AGA (anti-gliadin) and AEA (anti-endomysial) antibodies in the IgA/IgG class • In positive or symptomatic cases—small intestine biopsy	• Coeliac disease confirmed in 18 people (6.3%). • 94% of patients were AEA positive, 78% were AGA positive. • 15/18 patients had clinical symptoms (mainly intestinal), 3 cases were asymptomatic. • The authors recommend routine screening for coeliac disease in children and adolescents with DS.
Book, L., Hart, A., Black, J., Feolo, M., Zone, J.J., Neuhausen, S.L. (2001) [13]	97 children with DS (USA), Caucasian, age not specified (1–18 years)	Assessment of the prevalence and clinical characteristics of coeliac disease in children with DS in the USA	• Serological tests: IgA EMA antibodies • HLA DQA1/DQB1 genotyping • Clinical evaluation (intestinal symptoms, growth) • Analysis of first-degree relatives	• 10.3% of children with DS were EMA positive (probable coeliac disease). • Clinical symptoms were non-specific; only flatulence was more common in EMA+. • 88% of EMA+ children had a high risk of the HLA DQA10501 DQB10201 haplotype. • 10% of first-degree relatives of EMA+ children, also had EMA+. • The authors recommend routine screening for coeliac disease in children with DS.
Frazier, J.B., Friedman, B. (1996) [14]	19 children with DS (retrospective study)	Assessment of swallowing function and aspiration risk in children with DS	• Retrospective analysis of feeding behavior • Assessment of swallowing phases (oral, pharyngeal) • Observation of aspiration symptoms (coughing, choking)	• Pharyngeal phase disorders were found in 16/19 children. • Aspiration occurred in 10 children, often in the form of “silent aspiration” (asymptomatic). • Oral hypersensitivity made it difficult to accept foods with a specific texture. • Aspiration was considered a significant risk factor for respiratory diseases in children with DS.
Stanley, M.A., Shepherd, N., Duvall, N., Jenkinson, S.B., Jalou, H.E., Givan, D.C., … Roper, R.J. (2019) [15]	174 infants with Down syndrome, aged 0–6 months.	Assessment of the prevalence of feeding and swallowing disorders and risk factors for dysphagia in the youngest children with DS	• Retrospective analysis of medical records • Clinical assessment of feeding and swallowing problems • Videofluoroscopic Swallow Study (VFSS) • Analysis of risk factors (CHD, prematurity, low birth weight, desaturation, respiratory abnormalities)	• Dysphagia (oral and/or pharyngeal) was found in 55% of infants, and 39% required thickening of food or parenteral nutrition. • Highest risk of dysphagia: desaturation during feeding (OR = 15.8), respiratory abnormalities (OR = 7.2). • Prematurity and low birth weight increased the risk of dysphagia; severe heart defects were not significantly associated.
Białek-Dratwa, A., Żur, S., Sokal, A., Staśkiewicz-Bartecka, W., Kowalski, O. (2025) [16]	310 children, adolescents and young adults with DS in Poland (4 months–25 years)	Assessment of feeding difficulties and food neophobia and their associations with age, gender, and body weight	• CAWI survey (parents/guardians) • Montreal Children’s Hospital Feeding Scale (MCH-FS) • Food Neophobia Scale for Children (FNSC) • BMI analysis according to centile charts for DS • KomPAN—frequency questionnaire	• 26.5% had moderate to very severe feeding difficulties. • 41.3% showed high levels of food neophobia (most commonly in preschool age). • Neophobia and feeding difficulties were significantly correlated with age, but not with gender or body weight. • Children with high neophobia were less likely to consume vegetables, fish, legumes, nuts, wholemeal bread and red meat.
Ross, C.F., Bernhard, C.B., Smith-Simpson, S. (2019) [17]	157 children with DS (various ages; parental reports)	Assessment of the ease and difficulty of eating foods with different textures as perceived by parents	• Open questionnaire (parents indicated easy/difficult textures) • Qualitative analysis—categorization into 26 texture groups	• Most difficult textures: chewy, hard. • Easiest textures: creamy, crispy, soluble, mushy, pureed, smooth, soft. • With age, “easy” textures became crispy/dry/hard, and “difficult” textures became juicy. • The results emphasize the need to gradually expand the acceptance of textures in children with DS.
Connolly, B.H., Michael, B.T. (1986) [18]	24 children with intellectual disabilities: 12 with Down syndrome, 12 without DS; age 7.6–11 years (comparable mental age)	Assessment of motor skills (gross and fine)	Bruininks–Oseretsky Test of Motor Proficiency	• Children with DS scored lower than the control group in the following areas: running speed, balance, strength, and visual-motor control. • Girls with DS performed particularly poorly in strength, speed, manual dexterity and visual-motor coordination. • Both the composite scores for gross motor skills and fine motor skills were significantly worse in the DS group.
Virji-Babul, N., Kerns, K., Zhou, E., Kapur, A., Shiffrar, M. (2006) [19]	12 children with DS (8–15 years; adaptive age 3–7 years) vs. 12 TD (4–8 years)	Assessment of perceptual-motor abilities (motion recognition)	Tasks requiring differentiation of increasingly complex visual-motor cues	• Children with DS were able to recognize simple movement cues but had significant deficits in the perception of complex visual-motor cues. • Perceptual-motor deficits may hinder the development of motor skills and independence. • Conclusions: interventions should focus not only on “milestones” but also on improving the integration of perception and movement.
Fidler, Hepburn, Mankin, Rogers (2005) [20]	16 children with DS (mean age 33 months), 16 children with other developmental disabilities (matched age), 19 typically developing children	Assessment of motor skills and praxia in young children with DS and their relationship to adaptive functioning	Vineland Adaptive Behavior Scales, Mullen Scales of Early Learning, praxia task battery, object retrieval task	• Children with DS had significantly lower motor and praxia scores than the group with other developmental disabilities. • Specific praxia deficits were found (problems with hand and whole-body coordination, ineffective reaching strategies). • Praxia deficits correlated with daily functioning (Vineland Daily Living Skills). • Children with DS more often asked for help with tasks, indicating different compensatory strategies.
Białek-Dratwa A., Żur S., Wilemska-Kucharzewska K., Szczepańska E., Kowalski O. (2022). [21]	195 parents/caregivers of children, adolescents and young adults with DS (aged 0.5–30 years; median age 4.5 years; 32% of nursery age, 33% of preschool age, 12% of school age, 18% of teenage age, 4% of adult age)	Assessment of eating habits and potential dietary mistakes made by parents/caregivers	The study was conducted using the CAWI method with a validated questionnaire (30 questions) covering demographic data, comorbidities, feeding methods and eating habits of children with Down syndrome.	• Nutritional status: 21% underweight, 15.3% overweight, 5.6% obese. • Comorbidities: 52.3% hypothyroidism, 9.7% lactose intolerance, 3.6% coeliac disease, 3.6% Hashimoto’s disease. • Dietary eliminations: 62.6% no eliminations, 26.2% gluten elimination, 28.7% lactose elimination, 13.3% sucrose elimination, 8.7% casein elimination. • Early nutrition: 67.2% of children were breastfed, most often for 7–12 months. • Eating habits: vegetables and fruit—most often once a day (42.6% and 44.6%, respectively); dairy products—daily 36.9%, but 20% did not consume them at all; meat

## Data Availability

No new data were created or analyzed in this study.
